# Astragalus Polysaccharide Suppresses Skeletal Muscle Myostatin Expression in Diabetes: Involvement of ROS-ERK and NF-**κ**B Pathways

**DOI:** 10.1155/2013/782497

**Published:** 2013-12-18

**Authors:** Min Liu, Jian Qin, Yarong Hao, Min Liu, Jun Luo, Tao Luo, Lei Wei

**Affiliations:** ^1^Department of Pathology and Pathophysiology, School of Medicine, Wuhan University, Wuhan 430071, China; ^2^Central Laboratory, Renmin Hospital of Wuhan University, Wuhan 430060, China; ^3^Department of Cardiology, Renmin Hospital of Wuhan University, Wuhan 430060, China; ^4^Department of Anesthesiology, Renmin Hospital of Wuhan University, Wuhan 430060, China; ^5^Department of Pathology, Zhongnan Hospital of Wuhan University, Wuhan 430071, China

## Abstract

*Objective*. The antidiabetes drug astragalus polysaccharide (APS) is capable of increasing insulin sensitivity in skeletal muscle and improving whole-body glucose homeostasis. Recent studies suggest that skeletal muscle secreted growth factor myostatin plays an important role in regulating insulin signaling and insulin resistance. We hypothesized that regulation of skeletal muscle myostatin expression may be involved in the improvement of insulin sensitivity by APS. *Methods*. APS was administered to 13-week-old diabetic KKAy and nondiabetic C57BL/6J mice for 8 weeks. Complementary studies examined APS effects on the saturated acid palmitate-induced insulin resistance and myostatin expression in C2C12 cells. *Results*. APS treatment ameliorated hyperglycemia, hyperlipidemia, and insulin resistance and decreased the elevation of myostatin expression and malondialdehyde production in skeletal muscle of noninsulin-dependent diabetic KKAy mice. In C2C12 cells in vitro, saturated acid palmitate-induced impaired glucose uptake, overproduction of ROS, activation of extracellular regulated protein kinases (ERK), and NF-**κ**B were partially restored by APS treatment. The protective effects of APS were mimicked by ERK and NF-**κ**B inhibitors, respectively. *Conclusion*. Our study demonstrates elevated myostatin expression in skeletal muscle of type 2 diabetic KKAy mice and in cultured C2C12 cells exposed to palmitate. APS is capable of improving insulin sensitivity and decreasing myostatin expression in skeletal muscle through downregulating ROS-ERK-NF-**κ**B pathway.

## 1. Introduction

Skeletal muscle comprises 40% to 50% of the total body mass and is the major tissue responsible for insulin-dependent glucose utilization [1]. Impaired insulin-stimulated muscle glycogen synthesis plays a significant role in insulin resistance and noninsulin-dependent diabetes mellitus [2]. Several factors including elevated oxidative stress, inflammation, and free fatty acids have been implicated as the major defects responsible for causing muscle insulin resistance in patients with type 2 diabetes.

Myostatin is a growth factor produced by skeletal muscle and secreted into the circulation that negatively regulates muscle mass [3]. Recent studies suggest that myostatin plays a major role in regulating insulin signaling and insulin resistance [4]. Patients with type 2 diabetes had higher levels of muscle myostatin mRNA content than the control subjects [5]. A loss-of-function mutation in either one or both alleles of the myostatin gene was able to protect mice against obesity-induced insulin resistance [6]. Therefore, myostatin could be a potent target for the treatment of diabetes and insulin resistance.

The dry roots of *Astragalus membranaceus* (Fisch.) Bge. (Leguminosae), also known as Huang Qi in China, have long been used as an important component of herbal prescriptions to treat diabetes in traditional Chinese medicine [7–9]. Studies from our group and others have previously shown that astragalus polysaccharide (APS), the extracts of *Astragalus membranaceus*, can effectively alleviate diabetes and diabetes related complications such as cardiovascular and kidney disease [10–12]. One of the mechanisms of the APS's antidiabetic effect is to improve whole-body glucose homeostasis and increase insulin sensitivity in skeletal muscle [13]. We hypothesized that regulation of skeletal muscle myostatin expression may be involved in the improvement of insulin sensitivity by APS. In the current study, we investigated the effects of APS on myostatin expression in skeletal muscle of type 2 diabetic KKAy mice in vivo and culture skeletal muscle myocyte in vitro.

## 2. Materials and Methods

### 2.1. Chemicals and Reagents

*Astragalus membranaceus* (Fisch) Bunge var. mongholicus (Bunge) Hsiao was purchased from Shanghai Medicinal Materials (Shanghai, China). Identification was carried out by the Department of Authentication of Chinese Medicine, Hubei University of Chinese Traditional Medicine (Wuhan, China). APSs were extracted with optimized techniques using direct water decoction, as previously described [10]. C2C12 myoblast cell line was obtained from CCTCC (Wuhan, China). Insulin, 2-deoxyglucose, fatty acid-free bovine serum albumin (BSA), palmitate (PA), PD98059, and parthenolide were purchased from Sigma-Aldrich (Shanghai, China). High glucose-DMEM, fetal bovine serum (FBS), and horse serum were from GIBCO (Shanghai, China). NF-*κ*Bp65, I*κ*B*α*, ERK (42/44), phospho-ERK (42/44), p38, phospho-p38, SAPK/JNK, phospho-SAPK/JNK, and GAPDH antibodies were purchased from Cell Signal Technology (USA). Myostatin (GDF-8) antibody was purchased from Santa Cruz. Fluorescent-conjugated secondary antibodies were from LI-COR Biosciences.

### 2.2. In Vivo Experiments

The experimental procedures and protocols used in this investigation were approved by the Ethical Committee for the Experimental Use of Animals at Renmin Hospital of Wuhan University (Wuhan, China). Eight-week-old KKAy male mice and age-matched C57BL/6J male mice were purchased from Beijing HFK Bioscience Co. Ltd. (Beijing, China). Mice were housed under a 12 h light-dark cycle and an ambient temperature of 22°C. The KKAy mice were fed with a purified high-fat diet consisting of a percentage of total kcal of 41% fat, 41% carbohydrates, and 18% protein. The C57BL/6J mice were fed with a normal chow diet consisting of 12% fat, 60% carbohydrates, and 28% protein. KKAy mice were treated with either vehicle (KKAy, *n* = 8) or APS (KKAy + APS, *n* = 8), starting at 13 weeks of age. Age-matched C57BL/6J mice were also dosed with vehicle (C57BL/6J, *n* = 8) or APS (C57BL/6J + APS, *n* = 8) as healthy nondiabetic control animals. The APS was delivered slowly into the animal stomach through a stainless steel ball-tipped gavage needle at a dose of 700 mg kg^−1^ day^−1^ for 8 weeks. The control groups received an equal volume of vehicle (saline).

### 2.3. Blood Chemistry Assay

Blood glucose and insulin levels were determined before (age of 12 weeks) and after APS treatment (age of 20 weeks). Blood glucose levels were assessed using blood collected from the tail vein with a One-Touch Ultra blood glucose meter (LifeScan, Milpitas, CA, USA). Plasma insulin concentrations were determined using blood collected from the orbita of anesthetized animal following a 12 h overnight fasting period, with mouse high range insulin ELISA kit (ALPCO Diagnostics, USA). The index of homeostasis model assessment of insulin resistance (HOMA) was calculated as fasting plasma glucose [mmol/L] × fasting plasma insulin [mU/L]/22.5. Blood plasma FFAs were measured using the spectrophotometric NEFA C kit (Wako Chemicals, Neuss, Germany).

### 2.4. Malondialdehyde (MDA) Analysis in Skeletal Muscle

MDA level in the skeletal muscle was determined by the thiobarbituric acid (TBA) method with an assay kit according to manufactory guidance (Jiancheng Bioengineering Institute, Nanjing, China). Briefly, samples were incubated with TBA and SDS at 95°C for 1 h, followed by a centrifugation at 800 ×g for 10 min. Supernatants were transferred to a 96-well plate and the absorbance was measured at 532 nm. The MDA level after the calculation was further corrected by sample protein concentration (mmol/mg protein).

### 2.5. Cell Culture and Treatment

C2C12 myoblasts were cultured in Dulbecco's modified Eagle's medium (DMEM) supplemented with 10% FBS, 2 mM glutamine, 100 unit/mL penicillin, and 100 *μ*g/mL streptomycin. Cells were maintained at 37°C under 5% CO_2_ in a humidified incubator. Differentiation of myoblasts into myotubes was induced when the cells had achieved 70% confluence by replacing the media with DMEM containing 2% horse serum. Six days later, the differentiated C2C12 cells had fused into myotubes and were used for the experiments.

Palmitate (C16:0; Sigma) stock solutions of 20 mmol/L were dissolved in ethanol. Before application to the cells, palmitate was conjugated to bovine serum albumin (BSA) by diluting the palmitate solution with differentiation medium containing 5% (w/v) palmitate-free BSA (Sigma). Myotubes were incubated for 24 h in DMEM containing 5% BSA in either the presence (palmitate-treated cells) or absence (control cells) of 0.5 mmol/L palmitate. Cells treated with APS were incubated with additional APS at the final concentration of 200 *μ*g/mL.

### 2.6. In Vitro Glucose Uptake Assay

Glucose uptake was assayed using [3H]2-deoxyglucose according to previous report. After 24 h of treatment, cells were incubated in the presence or in the absence of 100 nm insulin for 30 min and then washed two times with wash buffer [20 mm HEPES (pH 7.4), 140 mm NaCl, 5 mm KCl, 2.5 mm MgSO_4_, and 1 mm CaCl_2_]. Cells were then incubated in buffer transport solution (wash buffer containing 10 *μ*M of unlabeled 2-deoxy-D-glucose and 0.5 *μ*Ci/mL [3H]2-deoxy-D-glucose) for 10 min. Nonspecific uptake was determined incubating the cells in the presence or in the absence of 5 *μ*M cytochalasin B. Uptake was terminated by aspiration of the solution. Cells were then washed three times, and radioactivity associated with the cells was determined by cell lysis in 0.05 m NaOH, followed by scintillation counting.

### 2.7. Measurement of ROS Production

C2C12 cells (2 × 10^5^ cells/well) in a 96-well plate were treated with or without APS for 1 h, followed by incubation with palmitate for 24 h. ROS generation was measured by incubation of the cells with 10 mM DCFH2-DA for 45 min. The fluorescence, corresponding to intracellular ROS, was measured on a Victor3 1420 Multilabel Counter (PerkinElmer, Turku, Finland) at 485 nm excitation and 530 nm emission wavelengths.

### 2.8. Measurements of mRNA

Total RNA was extracted from soleus muscle biopsies of both KKAy and C57BL/6J mice using TRIZOL reagent (Invitrogen) following the manufacture's instruction. Levels of myostatin mRNA were examined using semiquantitative reverse transcriptase (RT) PCR. One microgram of total RNA was reverse-transcribed with First Strand cDNA Synthesis Kit (Thermo Scientific). The sequences of primers used for amplification were 5′-GGTCTGCTGAGTTAGGAGGGT-3′ and 5′-TGTGTGTGTGGAGATGCACCT-3′. Amplification of gene yielded a single band of the expected size 339 bp. Thermal amplifications were carried out with PCR Master Mix (Thermo Scientific). PCR was performed within a linear range of amplification, which was determined in a preliminary experiment. All PCR data were normalized to beta-actin gene expression.

### 2.9. Western Blot Analysis

For the preparation of total protein extract, frozen tissues (50 mg) from soleus muscles or cells (1 × 10^6^) were homogenized in 0.5 mL or 0.2 mL ice-cold lysis buffer (50 mM Tris-HCl, pH 7.4, 1% NP-40, 0.25% sodium deoxycholate, 150 mM NaCl, 1 mM EGTA) containing protease inhibitor cocktail (1 mM phenylmethylsulfonyl fluoride, 1 *μ*g/mL aprotinin, 5 *μ*g/mL leupeptin, 1 mM Na_3_VO_4_, and 1 mM NaF). Tissue or cell lysates were centrifuged at 12,000 ×g for 15 min at 4°C. The supernatant was collected and stored at −70°C as total protein samples. The preparation of cytoplasmic and nuclear extracts was performed using a commercial kit (Pierce, USA) according to the manufacturer's instructions. Protein concentration was then determined using BCA protein assay kit. Samples of the lysates were separated by 10% SDS-PAGE and then transferred onto PVDF membranes. After being placed in blocking buffer, the membranes were incubated with the following primary antibodies (1 : 1,000 dilutions): anti-GDF-8, anti-NF-*κ*Bp65, anti-I*κ*B*α*, anti-p-P38, anti-P38, anti-p-ERK, and anti-ERK, anti-p-SARP/JNK, anti-SARP/JNK and GAPDH. Then, secondary antibody was conjugated to a fluorescent entity: IRDye 800-conjugated goat anti-rabbit IgG and/or Alexa Fluor 680-conjugated goat anti-mouse IgG (dilution 1 : 10000) in 10 mL LI-COR blocking buffer with gentle agitation for 1 h at room temperature. The membrane was scanned and analyzed on the Odyssey Infrared Imaging System (LI-COR Biosciences).

### 2.10. Statistical Analysis

Data are expressed as means ± SD. Statistical significance was determined using analysis of variance (ANOVA) followed by Tukey's test. A *P* value of less than 0.05 was considered statistically significant.

## 3. Results

### 3.1. Effect of APS on Body Weight, Blood Glucose, HOMA Score, and Plasma FFAs

**Figure 1:**
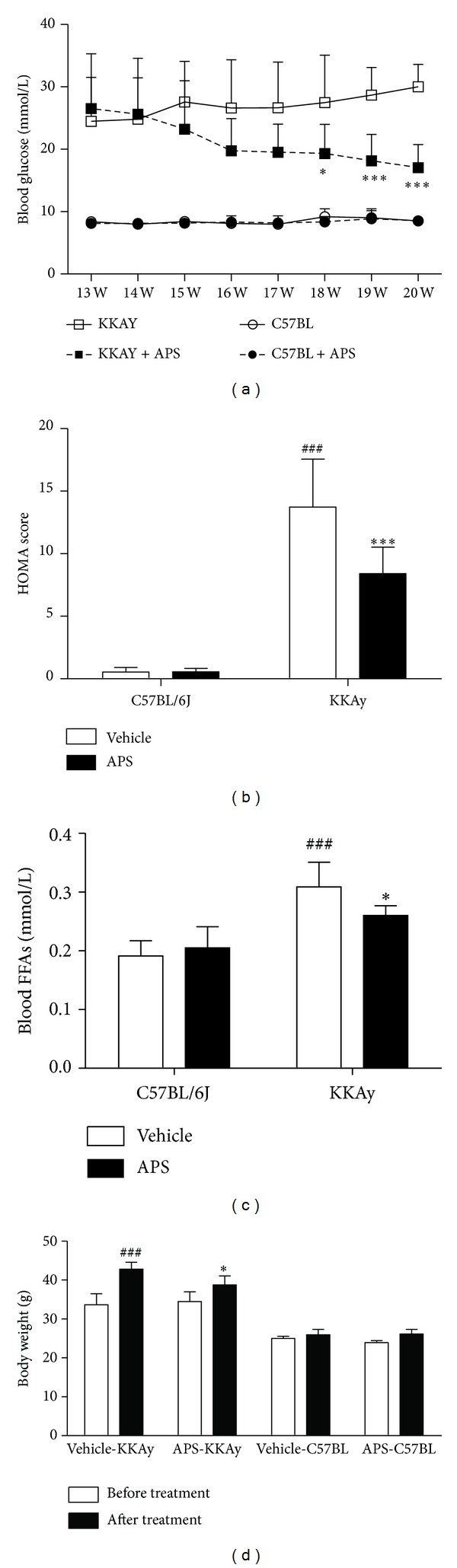
Effect of APS on blood glucose level (a), insulin resistance index HOMA score (b), plasma FFAs level (c), and body weight (d). Changes in blood glucose and body weight were evaluated before and after APS treatment for 8 weeks. Insulin resistance HOMA score and plasma FFAs were examined at the end of 8-week APS administration. Results are expressed as means ± SD for 8 animals in each group. ^###^ *P* < 0.001 versus C57BL/6J at the same age, *P < 0.05, ***P < 0.01 versus KKAy at the same age. HOMA: homeostasis model assessment; FFA: free fatty acids.

To test whether APS improves glucose metabolism and lowers insulin resistance in KKAy mice, APS was administered for 8 weeks starting at 13 weeks of age. During this period, blood was collected from the tail vein weekly and plasma glucose levels were measured. Consistent with our previous finding, plasma glucose was significantly reduced in the APS + KKAy group compared with the KKAy group. In particular, at the end of 8 weeks of APS treatment, the glucose level was 30 ± 3.63 mM in the KKAy group and approximately 17.05 ± 3.69 mM in the APS + KKAy group (*P* < 0.001, *n* = 8, Figure 1(a)).

At the beginning of APS treatment, the index of homeostasis model assessment of insulin resistance (HOMA Score) was not different between the APS + KKAy group and the KKAy group (data not shown). However, after 8 weeks treatment, the HOMA score (Figure 1(b)) was significantly lower in the APS + KKAy (8.41 ± 2.12) group than in the KKAy group (13.72 ± 3.84, *P* < 0.001, *n* = 8). Similarly, APS treatment resulted in a significant decrease in plasma FFA levels (Figure 1(c)) and weight gain (Figure 1(d)) for the KKAy mice (*P* < 0.05, KKAy + APS versus KKAy at the age of 20 weeks), whereas APS by itself had no effect on body weight and glucose metabolism in nondiabetic C57BL/6J mice.

### 3.2. Effect of APS on MDA Production and Myostatin Expression in KKAy Mice

**Figure 2:**
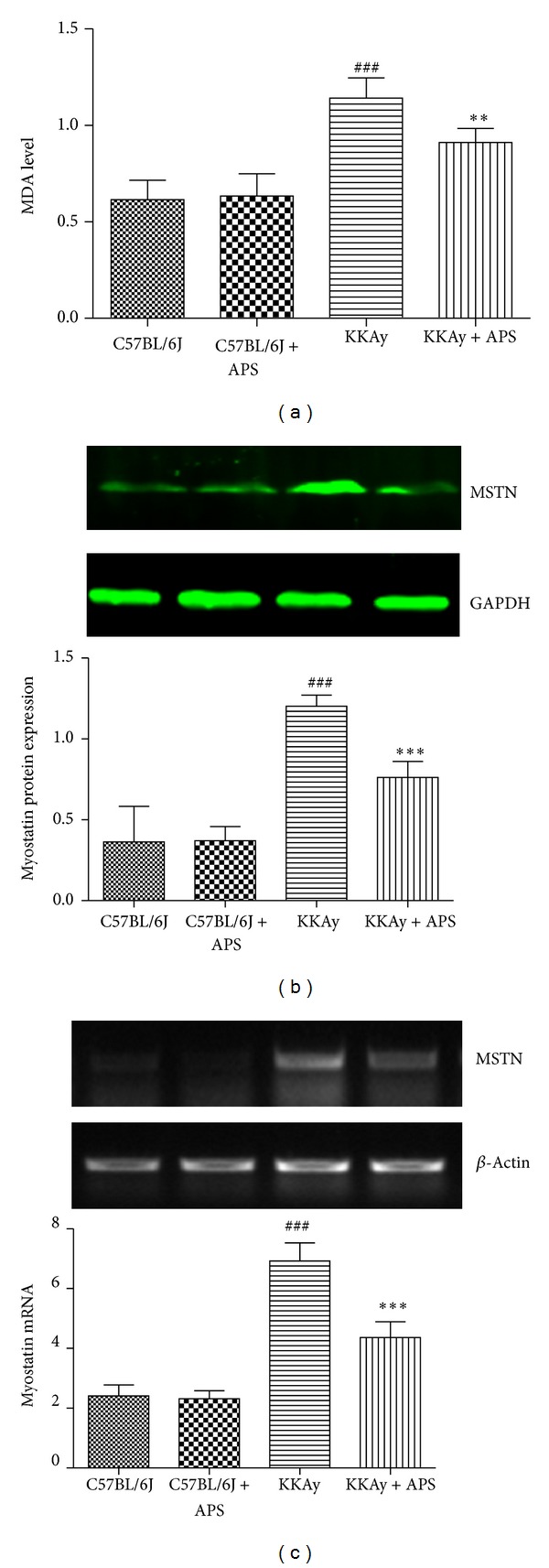
Effect of APS on MDA production and myostatin expression in skeletal muscle. At the end of 8-week APS administration, skeletal muscle MDA level (a), myostatin protein (b), and mRNA expression (c) were assessed, respectively. Results are expressed as means ± SD for 6 animals in each group. ^###^ *P* < 0.001 versus C57BL/6J, **P < 0.01, ***P < 0.001 versus KKAy. MDA: malondialdehyde; MSTN: myostatin.

Oxidative stress plays a causal role in the development of insulin resistance. We tested whether APS treatment alters skeletal muscle redox balance by assessing MDA levels, a stable indicator of oxidative stress. As shown in Figure 2(a), MDA level in the skeletal muscle of KKAy mice was increased approximately 2-fold when compared with that in the C57BL/6C mice (*P* < 0.001, *n* = 6, KKAy versus C57BL/6J). APS administration significantly decreased muscular MDA content (*P* < 0.01, *n* = 6, KKAy + APS versus KKAy).

Since myostatin is expressed predominantly in skeletal muscle, we therefore set out to determine the effects of the APS on both protein and mRNA levels of myostatin in the skeletal muscle tissues of KKAy mice. Immunoblotting assay showed that there was a 3.3-fold increase in the levels of myostatin in KKAy mice (*P* < 0.001, *n* = 6, KKAy versus C57BL/6J). APS administration for 8 weeks resulted in 37% decrease in myostatin as compared to vehicle treated KKAy group (*P* < 0.001, *n* = 6, KKAy + APS versus KKAy) (Figure 2(b)).

The decreased levels of myostatin could be due to the decrease in generation or increase in degradation of myostatin. We then assessed the effects of the APS on the mRNA levels of myostatin (Figure 2(c)). Compared with normal C57BL/6J mice, diabetic KKAy mice exhibited prominent upregulated myostatin gene expression*  * (*P* < 0.001, *n* = 6, KKAy versus C57BL/6J) in the skeletal muscle tissues. The increases in myostatin mRNA were significantly attenuated by APS treatment (*P* < 0.001, *n* = 6, KKAy + APS versus KKAy).

### 3.3. APS Attenuates Insulin Resistance in C2C12 Cells In Vitro

**Figure 3:**
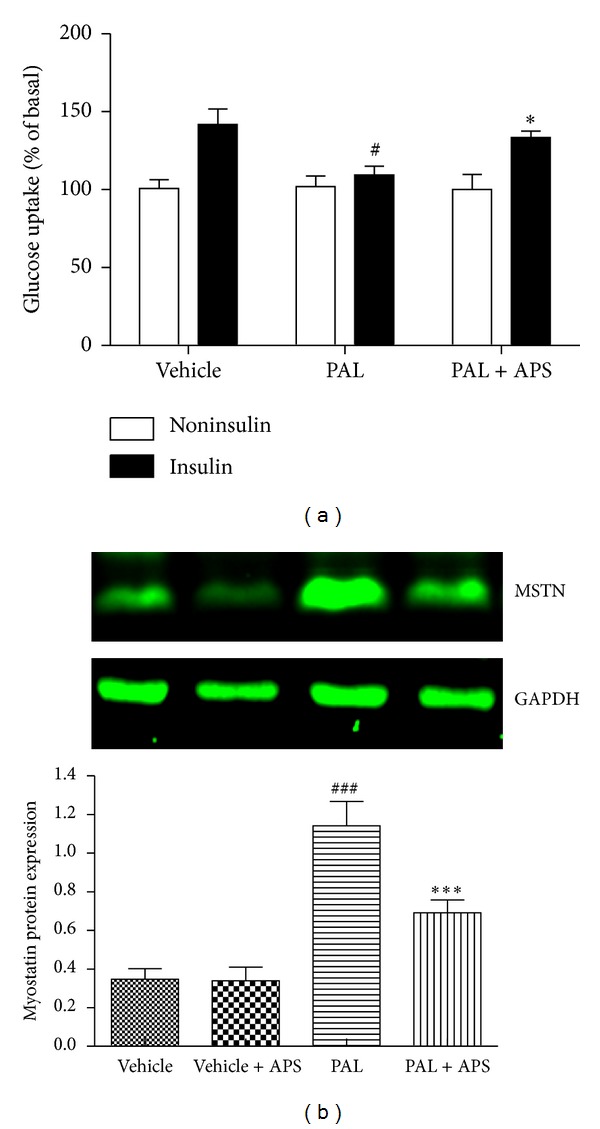
Effect of APS on glucose uptake and myostatin expression in C2C12 cells exposure to palmitate. C2C12 cells were incubated for 24 h in DMEM containing 5% BSA in either the presence (FFA-treated cells) or absence (control cells) of 0.5 mmol/L FFAs. Cells treated with APS were incubated with additional APS at the final concentration of 200 *μ*g/mL. (a) Glucose uptake was assayed using [3H]2-deoxyglucose in the presence or in the absence of 100 nM insulin. ^#^ *P* < 0.05 versus vehicle, *P < 0.05 versus palmitate. (b) Myostatin protein expression was assessed by western blot. Data are expressed as means ± SD of 6 different experiments. ^###^ *P* < 0.001 versus vehicle, ***P < 0.001 versus palmitate; PAL: palmitate; MSTN: myostatin.

Elevated lipids can cause insulin resistance, and exposure of C2C12 skeletal muscle cells to the FFA palmitate has been widely used as an in vitro model of insulin resistance. To extend these observations from KKAy mice to a cellular model of insulin resistance, C2C12 cells were treated with palmitate to induce insulin resistance, as assessed by their decreased intracellular glucose uptake. C2C12 cells exposed to 0.5 mmol/L palmitate showed a 23% (*P* < 0.05) reduction in insulin-stimulated deoxyglucose uptake compared with untreated control skeletal muscle cells (Figure 3(a)). This palmitate-induced insulin resistance coincided with a 3.3-fold increase in myostatin accumulation (Figure 3(b)). As expected, APS administration resulted in significant reduction of myostatin expression and improvement in insulin-stimulated deoxyglucose uptake.

### 3.4. APS Attenuates Palmitate-Induced Generation of ROS in C2C12 Cells

**Figure 4:**
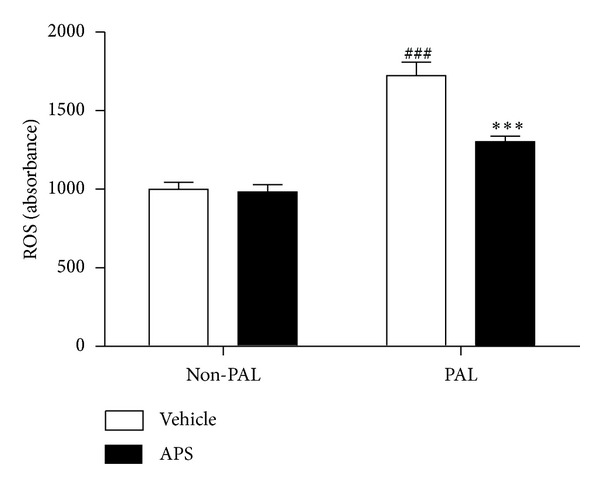
Effects of APS on the production of ROS in palmitate-stimulated C2C12 cells. C2C12 cells were incubated for 24 h in DMEM containing 5% BSA in either the presence (FFA-treated cells) or absence (control cells) of 0.5 mmol/L FFAs. Cells treated with APS were incubated with additional APS at the final concentration of 200 *μ*g/mL. The intracellular levels of ROS were determined by fluorescence using a fluorescence microplate reader with excitation/emission set to 485/530 nm. Cells were plated at 1 × 10^6^ cells/mL and pretreated with APS for 0.5 h, followed by incubation with palmitate for 24 h. Data are expressed as means ± SD of 6 different experiments. ^###^ *P* < 0.001 versus vehicle, ***P < 0.001 versus palmitate. ROS: reactive oxygen species. PAL: palmitate.

Oxidative stress has been implicated in the pathogenesis of insulin resistance. It is suggested that increased ROS levels are an important trigger for insulin resistance. We next investigated whether ROS levels were altered by palmitate and APS treatment. As shown in Figure 4, ROS levels were increased in C2C12 cells by exposure to palmitate (0.5 mmol/L, 24 h). APS treatment significantly inhibited generation of ROS in response to palmitate.

### 3.5. APS Inhibits ERK/NF-*κ*B Pathway in C2C12 Cells

**Figure 5:**
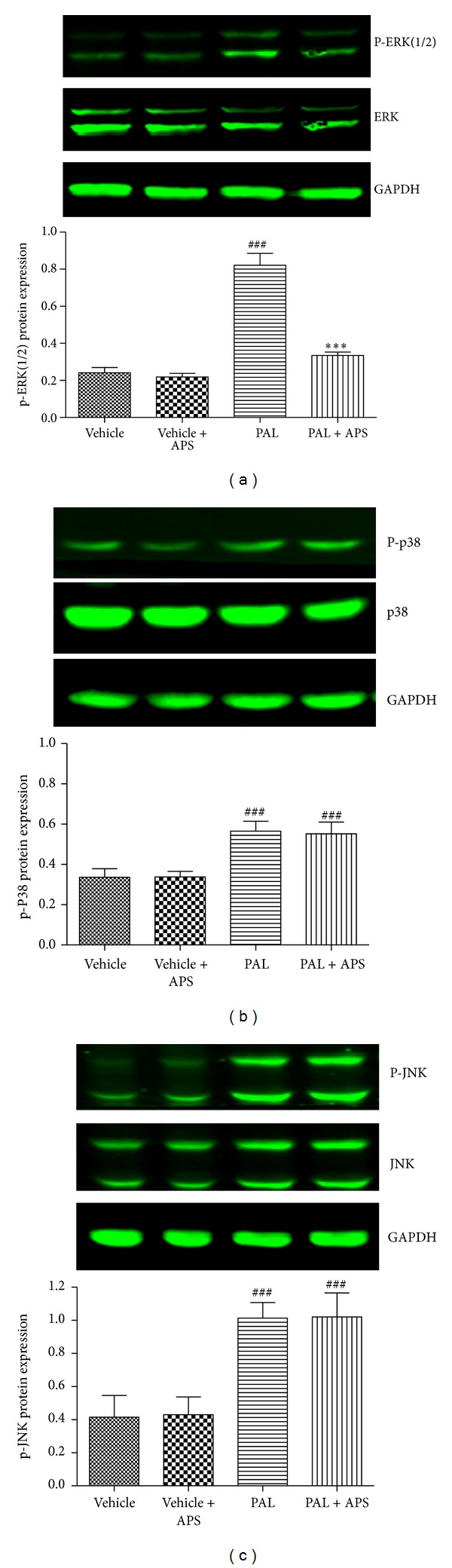
Effects of APS on the MAPK expression in palmitate-stimulated C2C12 cells. C2C12 cells were incubated for 24 h in DMEM containing 5% BSA in either the presence (FFA-treated cells) or absence (control cells) of 0.5 mmol/L FFAs. Cells treated with APS were incubated with additional APS at the final concentration of 200 *μ*g/mL. The expression of ERK, p38, and JNK and phosphorylated ERK, p38, and JNK were determined by western blot. Data are expressed as means ± SD of 6 different experiments. ^###^ *P* < 0.001 versus vehicle, ***P < 0.001 versus palmitate. ERK: extracellular signal-regulated kinase; JNK: c-Jun amino-terminal kinases.

ROS have been shown to induce various signaling pathways, including the p38 MAPK, ERK, and JNK pathways. We then investigated the downstream pathways involved in the upregulation of myostatin following palmitate exposure. Immunoblotting detection of total and phosphorylated ERK1/2 (Figure 5(a)) revealed that palmitate treatment induced activation of phosphorylated ERK1/2 (3.4-fold induction, *P* < 0.001). The upregulation of phosphorylated ERK1/2 levels achieved by palmitate was abrogated when the cells were coincubated with APS (*P* < 0.001 versus palmitate-treated cells, Figure 5(a)). Although palmitate treatment also induced phosphorylation of P38 and JNK, APS showed no significant effect on P38 and JNK activation. (Figures 5(b)-5(c)). These results indicate that inhibition of the MAPK-ERK cascade might be involved in the decrease of myostatin following APS treatment.

**Figure 6:**
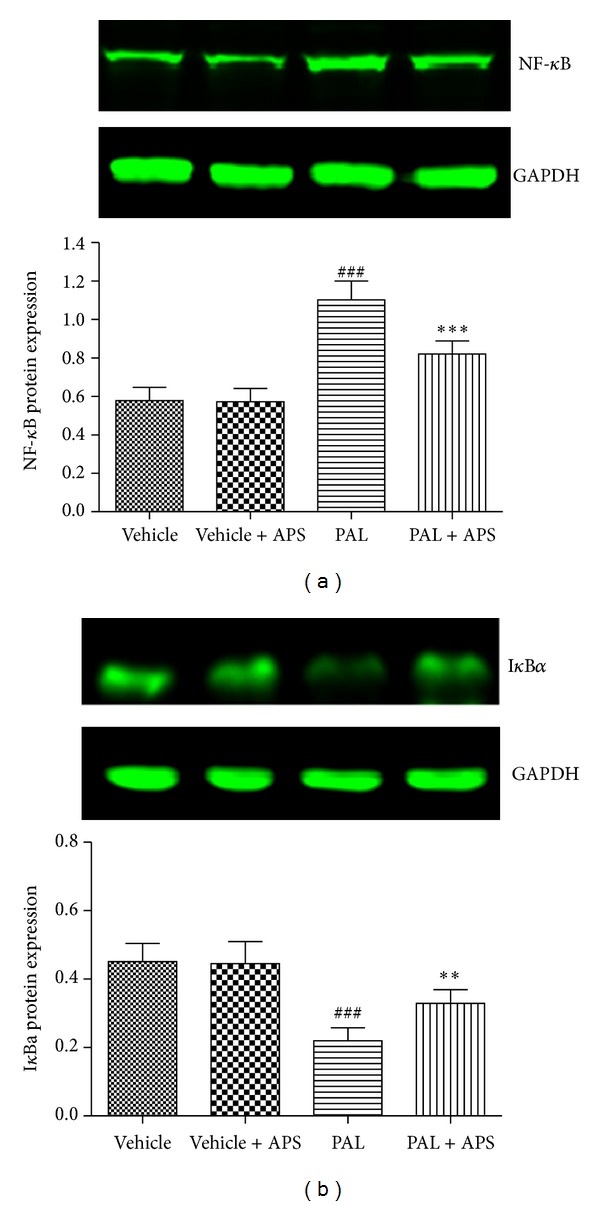
Effects of APS on NF-*κ*B activation in palmitate-stimulated C2C12 cells. C2C12 cells were incubated for 24 h in DMEM containing 5% BSA in either the presence (FFA-treated cells) or absence (control cells) of 0.5 mmol/L FFAs. Cells treated with APS were incubated with additional APS at the final concentration of 200 *μ*g/mL. The expression of nuclear NF-*κ*B and cytoplasmic I*κ*Ba was determined by western blot. Data are expressed as means ± SD of 6 different experiments. ^###^ *P* < 0.001 versus vehicle, **P < 0.01, ***P < 0.001 versus palmitate. NF-*κ*B: nuclear factor-kappa B.

It is reported that C2C12 exposure to palmitate activates NF-*κ*B and activation of the MAPK-ERK cascade may influence NF-*κ*B activation in skeletal muscle. We next determined whether activation of this transcription factor was involved in palmitate-mediated myostatin upregulation. Palmitate treatment resulted in a 1.9-fold increase in nuclear NF-*κ*Bp65 protein, whereas this activation was prevented in cells coincubated with palmitate and APS (Figure 6(a)). Since NF-*κ*B is located in the cytosol bound to the inhibitor I*κ*B, we next assessed whether palmitate resulted in changes in the content of I*κ*Ba (Figure 6(b)). Palmitate addition to cells caused a 51% decrease (*P* < 0.001) in the abundance of I*κ*Ba, whereas APS significantly blocked the palmitate-induced I*κ*Ba degradation (Figure 6(b)), thereby inhibiting activation and translocation of NF-*κ*B.

**Figure 7:**
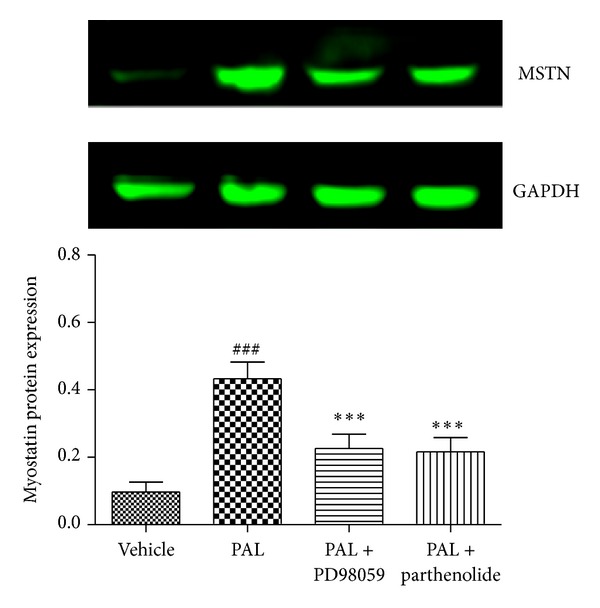
The ERK-MAPK cascade is involved in palmitate-induced myostatin expression in skeletal muscle cells. C2C12 myotubes were incubated with either 0.5 mM palmitate in the presence or in the absence of 100 *μ*M PD98059 or with 10 *μ*M parthenolide. Analysis of the protein levels of myostatin was analyzed by western blot assay. Data are expressed as means ± SD of 6 different experiments. ^###^ *P* < 0.001 versus vehicle, ***P < 0.001 versus palmitate. MSTN: myostatin.

To clearly demonstrate whether ERK1/2/NF-*κ*B pathway was involved in APS downregulating palmitate-induced myostatin expression, we used pharmacological inhibitors PD98059 and parthenolide to block ERK and NF-*κ*B, respectively. Coincubation of the cells with palmitate, in either the presence of PD98059 or parthenolide, prevented the upregulation of myostatin (Figure 7). Overall, these results suggest that myostatin downregulation in skeletal muscle cells following APS exposure is regulated by the ERK-MAPK-NF-*κ*B pathway.

## 4. Discussion

We showed that APS treatment ameliorated hyperglycemia, hyperlipidemia, and insulin resistance, which were associated with the decreased MDA and myostatin level in the skeletal muscle of KKAy mice. Myostatin is an important negative regulator of skeletal muscle growth. In our study, increased myostatin expression is inversely related to insulin sensitivity in diabetes. The finding suggests the concept that myostatin may play a role in the development of insulin resistance [14]. Previous report by Palsgaard et al. performed a gene chip analysis of skeletal muscle biopsies from human subject and demonstrated that the levels of myostatin mRNA were increased in type 2 diabetics [5]. The elevation of muscle and plasma myostatin protein contents in insulin-resistant patients can be decreased by aerobic exercise training [15]. Notably, preclinical study showed that administration of a neutralizing antibody to myostatin for six weeks can effectively reduce the blood glucose level in ob/ob mice [16], while injection of recombinant myostatin decreased insulin sensitivity in healthy male mice [15]. The mechanisms by which myostatin achieves this suppression of insulin sensitivity have not been clearly defined. Myostatin is known to increase glucose uptake and glycolysis and inhibit glycogen synthesis in cultured skeletal muscle cells in vitro via an AMP kinase-dependent mechanism [17]. Myostatin may also affect glucose uptake indirectly through its effects on TNF-*α* expression, which can antagonize the effects of insulin on glucose uptake [18].

Since elevated plasma FFA is a major cause of insulin resistance in type 2 diabetes [19], we therefore used an in vitro FFA-induced insulin resistance cell culture model to further characterize the mechanisms by which APS attenuating myostatin expression. In this in vitro model, FFA palmitate inhibited insulin-stimulated glucose uptake, accompanied with a strong induction of the myostatin expression protein in skeletal muscle C2C12 cells, which was significantly attenuated by APS. Because an inverse relationship between the insulin resistance and muscle myostatin has been reported [14], we hypothesized that reducing skeletal muscle myostatin expression might be one of the mechanisms by which APS enables insulinsensitizing and hypoglycemic activity.

Oxidative stress, which may be precipitated by hyperglycemia and hyperlipidemia, plays a pivotal role in the development of diabetes [20]. ROS overproduction can lead to impairment of intracellular signaling pathways and development of insulin resistance [21]. Oxidative stress has been proposed as a link between FFA and skeletal muscle insulin resistance [22]. Consequently, reducing oxidative stress by lowering ROS production is crucial in the management of insulin resistance. We showed that palmitate stimulates ROS formation and APS presented effective suppression on palmitate-induced ROS overproduction and facilitated insulin action on the C2C12 cells, well demonstrating its antioxidant potency against FFA insult. Consistent with our in vitro finding, the decreased MDA level in skeletal muscle of KKAy mice after APS treatment also supported an antioxidant action of APS. There are several pathways involved in ROS production under the conditions of insulin resistance, including NADPH oxidase, xanthine oxidase, and mitochondria-mediated pathways [23]. APS is reported to protect mitochondria by scavenging ROS, inhibiting mitochondrial permeability transition, and increasing the activities of antioxidases [24]. Future research will therefore involve investigations of the mechanisms behind the antioxidant effect of APS in response to palmitate stimulation and other pathophysiological conditions.

We studied several different signal transduction pathways known to be activated by ROS. MAPKs are important mediators involved in a variety of cell signalling functions, including insulin resistance. The MAPK family includes p38, ERK, and JNK [25]. Our data indicate that palmitate induced marked phosphorylation of ERK in C2C12 cells, while APS downregulated the expression of the phospho-ERK following palmitate stimulation. The transcription factor NF-*κ*B has been proposed as a critical mediator between oxidant stress and gene expression. Activation of the NF-*κ*B has been suggested to participate in diabetes and its complications [26]. We therefore explored whether NF-*κ*B participates in APS attenuating palmitate-induced myostatin expression and insulin resistance. Exposure of C2C12 cells to palmitate resulted in degradation of I*κ*B*α* and the subsequent release and translocation of NF-*κ*B into the nucleus. APS administration, however, leads to the upregulation of I*κ*B*α* and decreased NF-*κ*B translocation. We propose that APS may reduce FFA palmitate-stimulated myostatin expression in skeletal muscle cells through a mechanism involving activation of the ROS-ERK-NF-*κ*B pathway, since the ERK inhibitor PD98059, as well as NF-*κ*B inhibitor parthenolide, partially reversed the effect of palmitate-stimulated myostatin expression.

Several other lines of evidences also suggest an effect of APS on ROS-ERK/NF-*κ*B signaling pathway. For example, *Astragalus membranaceus* can inhibit inflammation via phospho-P38 MAPK and NF-*κ*B pathways in advanced glycation end product-stimulated macrophages [18]. *Astragalus membranaceus* has been shown to inhibit mRNA expressions of NF-*κ*B and I*κ*B in renal cortex of streptozotoxin-induced diabetic rats [27]. Similar to our finding, a recent study suggests that astragalus polysaccharide inhibits palmitate-induced insulin resistance in C2C12 myotubes by inhibiting expression of PTP1B and regulating NF-*κ*B [28]. In previous studies from our colleagues, APS has been shown to increase insulin-induced tyrosine phosphorylation of the insulin receptor and IRS-1 in the skeletal muscles of fat-fed diabetic rats, with parallel reduces in protein levels and activity of protein tyrosine phosphatase-1B [10]. Furthermore, our recent finding indicated that the antihyperglycemic activity of APS is mediated by insulin sensitivity improvement related to GSK3 inhibition in the liver [11]. All these results suggest that APS might regulate insulin sensitivity at multiple sites in various diabetic animal models.

In conclusion, the present study demonstrated elevated myostatin expression in skeletal muscle of type 2 diabetic KKAy mice and in cultured C2C12 cells exposed to palmitate. APS is capable of improving insulin sensitivity and decreasing myostatin expression in skeletal muscle through downregulating ROS-ERK-NF-*κ*B pathway. This study provides new insight into the molecular mechanisms of antidiabetes action of APS.
